# Attitudes of clinical staff toward the causes and management of aggression in acute old age psychiatry inpatient units

**DOI:** 10.1186/1471-244X-14-80

**Published:** 2014-03-19

**Authors:** Terence V McCann, John Baird, Eimear Muir-Cochrane

**Affiliations:** 1 Discipline of Mental Health Nursing and Aged Care, College of Health and Biomedicine, Victoria University, PO Box 1428, Melbourne 8001, Victoria, Australia; 2 NorthWestern Mental Health Old Aged Persons’ Mental Health Program, Harvester Building, 4C Devonshire Road, Sunshine 3020, Victoria, Australia; 3 School of Nursing and Midwifery, Flinders University, GPO Box 2100, Adelaide, South Australia 5001, Australia

**Keywords:** Aggression, Attitudes, Elderly, Old age psychiatry, Nurses, Restraint, Seclusion, Survey

## Abstract

**Background:**

In psychiatry, most of the focus on patient aggression has been in adolescent and adult inpatient settings. This behaviour is also common in elderly people with mental illness, but little research has been conducted into this problem in old age psychiatry settings. The attitudes of clinical staff toward aggression may affect the way they manage this behaviour. The purpose of this study was to examine the attitudes of clinical staff toward the causes and management of aggression in acute old age psychiatry inpatient settings.

**Methods:**

A convenience sample of clinical staff were recruited from three locked acute old age psychiatry inpatient units in Melbourne, Australia. They completed the Management of Aggression and Violence Scale, which assessed the causes and managment of aggression in psychiatric settings.

**Results:**

Eighty-five staff completed the questionnaire, comprising registered nurses (61.1%, n = 52), enrolled nurses (27.1%, n = 23) and medical and allied health staff (11.8%, n = 10). A range of causative factors contributed to aggression. The respondents had a tendency to disagree that factors directly related to the patient contributed to this behaviour. They agreed patients were aggressive because of the environment they were in, other people contributed to them becoming aggressive, and patients from certain cultural groups were prone to these behaviours. However, there were mixed views about whether patient aggression could be prevented, and this type of behaviour took place because staff did not listen to patients. There was agreement medication was a valuable approach for the management of aggression, negotiation could be used more effectively in such challenging behaviour, and seclusion and physical restraint were sometimes used more than necessary. However, there was disagreement about whether the practice of secluding patients should be discontinued.

**Conclusions:**

Aggression in acute old age psychiatry inpatient units occurs occasionally and is problematic. A range of causative factors contribute to the onset of this behaviour. Attitudes toward the management of aggression are complex and somewhat contradictory and can affect the way staff manage this behaviour; therefore, wide-ranging initiatives are needed to prevent and deal with this type of challenging behaviour.

## Background

Aggression (‘any form of behaviour that is intended to injure someone physically or psychologically’ [1], p.6) occurs commonly in elderly people, aged 65 years and older, with mental illness and is mainly associated with dementia [2]; for instance, 15-43% of community referrals to old age psychiatry services are because of aggression [3]. Staff working in inpatient units for elderly people with organic mental illness are more likely to be to be assaulted than those working in other inpatient settings [4]. When aggression occurs in inpatient settings it is more likely to be directed at nurses than other patients [2,5], or at other clinical and non-clinical staff [4], and rarely culminates in severe injury [2,6,7]. Little research has been undertaken to examine the attitudes of staff toward patient aggression in acute old age psychiatry inpatient units. It is important to examine these attitudes because they may affect the way staff attempt to prevent and manage this behaviour. This paper adds to the literature on aggression by presenting the findings of a survey of clinical staff attitudes toward this behaviour in inpatient units for elderly people with mental health problems in Australia.

### Causes of aggression

The causes of aggression in elderly patients with mental health problems are complex and interrelated. Aggression may be attributable to psychosocial-environmental factors or the complex interaction of patients, staff and inpatient unit culture influences [8]. It may be due to the inability of elderly patients with dementia to communicate their needs effectively [9], poor staff-to-patient interactions or the environment of care [9,10], overcrowding, lack of privacy, lack of activities, weak clinical leadership [11], being denied something [7], and restricting patients’ freedom [12].

There is also some debate that aggression may be due to the elderly person having a pre-morbid personality trait of aggression. Various reviews and studies have concluded, however, that an inconclusive relationship exists between premorbid personality and this behaviour [13,14]. For instance, a systematic review conducted by Osborne, Simpson and Stokes [14] concluded that 72% of studies detected significant relationships between pre-morbid personality ─ particularly pre-morbid neuroticism ─ and challenging behaviour such as aggression; however, several studies found no relationship. In addition, there is a direct association between patients with Alzheimer’s disease who have a recent history of aggression [15] and childhood behavioural problems.

There are also contrasting reports about a relationship between particular illnesses and aggression. On the one hand, there are claims that aggression is associated with the person’s illness [9]. In particular, positive relationships have been reported between certain symptomatology, such as paranoid delusional thinking [5,16], impulsivity [17], dementia symptoms [2], depressive symptoms [13], and aggression. In contrast, James et al. [18] found no diagnosis was positively associated with aggression in an acute psychiatric ward.

### Restraint and seclusion

When confronted by aggression, clinical staff may use a range of person-centred (e.g., good staff-to-patient communication, distraction, de-escalation) and containment (e.g., restraint, seclusion, medication) measures to manage this behaviour. Two containment approaches ─ restraint and seclusion ─ are examined because there is debate about their use in the literature. Restraint (the restriction of a person’s freedom of movement by physical, mechanical, chemical, and/or emotional means [19]) and seclusion (the sole confinement of a person in a room where the doors and windows are locked [20]) may be used to prevent and manage aggression [21] but they can have adverse effects on elderly patients in old age psychiatry in particular. Both practises are contrary to prominent international recommendations [22], government reports, mental health service policies, service user organisations and scholarly literature, which advocate that as strategies to deal with disturbed behaviour, restraint and seclusion should be used as little as possible or eliminated.

International variation is evident in containment practises, and this is influenced by a range of factors such as concerns about efficacy and patient safety, and broader cultural values [23]. For instance, in the United Kingdom mechanical restraint is not used and is regarded by many nurses to be reprehensible [23]; in Finland, overall, seclusion is used more frequently than mechanical restraint, but there are regional variations in these practises [24]. In Australia, while there are no national data on the use of restraint and seclusion, the reduction and potential elimination of restraint and seclusion practises and adverse events have been a recommendation of the recent National Mental Health Commission [25], and have been identified as one of four key national priority areas for increasing safety and reducing harm in mental health care [26]. In acute old age psychiatry inpatient settings in particular, restraint use has come under increased examination as a consequence of evidence it may be deleterious [26], ineffective, and unnecessary [27]. The overuse of restraint and seclusion is regarded as an early sign of a mental health system under pressure [25].

Research into the use of restraint in elderly people has been conducted in general hospitals [28] and nursing home settings [9,10,29]. However, there has been little investigation of the use of these containment practices with elderly persons in old age psychiatry inpatient settings, and the rate and type of restraint use can vary, even within neighbouring units [30]. Furthermore, Cochrane reviews of restraint and seclusion [31] and containment practises [32] have concluded that non-pharmacological approaches to restraint and seclusion are not supported by evidence from controlled studies.

### Attitudes toward aggression

The attitudes of clinical staff toward aggression can influence the way they respond to this behaviour [33,34]. Positive attitudes may influence the adoption of person-centred approaches whereas negative attitudes may contribute to the use of containment measures. Studies undertaken in various settings have provided contradictory findings about the attitudes of staff toward patient aggression. A United Kingdom survey in a high secure hospital, by Pulsford et al. [35], highlighted that staff held a range of attitudes about the causes and management of patient aggression. However, a United Kingdom survey of the attitudes of staff toward aggressive older people with dementia in residential care, by Pulsford et al. [10], noted that this behaviour was attributable to interpersonal problems resulting from unfavourable situational events such as an adverse environment of care and poor interaction with others in the setting. Furthermore, the staff response to aggression was mainly to adopt a person-centred approach rather than measures such as restraint, seclusion and medication. In contrast, a survey of staff attitudes toward patient aggression in dementia facilities in Japan, by Nakahira et al. [36], reported a significant relationship between staff with negative attitudes toward aggressive patients and the use of physical and chemical restraint. Similarly, Duxbury & Whittington [37], in a United Kingdom survey of staff attitudes toward aggression in an acute psychiatric inpatient setting, highlighted that respondents who perceived internal influences on patients contributed to this behaviour, such as the nature of their illness, were more likely to use containment than person-centred methods to manage this behaviour. The implication of possessing negative attitudes toward aggression and adoption of containment measures is these approaches can culminate in adverse health outcomes for elderly patients in particular. For example, a survey and data set analysis of all nursing homes (N = 740) in Pennsylvania in the United States found a significant association between physical restraint use and subsequent deterioration in patients’ cognitive and activities of daily living performance and increased walking dependence [9,10,29].

Little research has been carried out into the attitudes of clinical staff toward the causes and management of patient aggression in psychiatry in general [33,34] and in acute old age psychiatry in particular [2]. In light of the relationship between attitudes toward aggression and the adoption of measures to deal with this behaviour, and the implications for the patient’s wellbeing, the purpose of this study was to examine the attitudes of clinical staff toward the causes and management of aggression in acute old age care psychiatry inpatient settings.

## Method

### Study design

A survey design was used, incorporating a structured questionnaire.

### Sample & setting

A convenience sample of clinical staff was recruited from three locked acute old age psychiatry inpatient units and their associated community outreach teams, in Melbourne, Australia. The units are managed by the same public mental health service, but each is located in separate geographical locations. The units offer mainly single-room accommodation with en suite toilets, and all have common recreational and dining facilities and gardens. Patients aged 65 years and over are admitted directly to the units from the community or residential care for short-term management of an acute phase of mental illness, until they recover enough to be treated in a community-based setting, and are different, therefore, from nursing homes that cater specifically for people with dementia. Medical, nursing and allied health staff provide the care, and the staffing ratios are broadly similar across the units.

The inclusion criterion for staff was clinical staff employees (Unit manager, Registered/enrolled nurses, psychologists, social workers, occupational therapists, psychiatrists) working on day shifts in the respective units. The exclusion criterion was staff working at night and at weekends. Staff received written invitations to participate, and the project was explained to them either at staff meetings or individually. They were given the choice to complete the questionnaire and hand it back to the researcher, or to return it by mail. Almost all chose the former option.

### Instrument

The Management of Aggression and Violence Scale (MAVAS) [37-39] was used to assess attitudes toward the causes of, and ways to manage, aggression. It originally contained 27 items, on a four-point Likert scale, ranging from 1 (strongly agree) to 4 (strongly disagree), with the cut-off for agreeing set at 2.5 (A 5-point version of the scale is also available). A low score indicates agreement with a statement. The Scale has undergone psychometric evaluation and has been shown to contain a strong four-factor structure: internal, external and situational/interactional influences on aggression [38]. (i) Internal (n = 5): Aggression is due mainly to factors within the aggressive person (e.g., mental illness or personality); (ii) External (n = 3): Aggression is caused mainly by influences in the person’s physical or social environment (e.g., physical layout of ward, or manner in which the ward is managed by staff); (iii) Situational/interactional (n = 5): Aggression is attributable to factors in the immediate environment, such as the way staff interact with patients; (iv) Approaches to the management of aggression (n = 14): (e.g., use of medications, restraint and seclusion). The most recent version of the instrument, which was used in the present study, contains 30 items; the three additional items focus on cultural/gender issues.

The internal consistency of the instrument has been established in several studies [38-40]. The Cronbach’s alpha in the present study was 0.8. Ideally, Cronbach’s alpha should be above 0.7 [41].

### Ethics

Ethical approval to carry out the study was obtained from Melbourne Health Mental Health Research and Ethics Committee. Researchers, who were not employees of the mental health service, undertook recruitment. Return of questionnaires was interpreted as consent.

### Statistical analysis

Data analyses were undertaken using the R environment for statistical computing and graphics [42]. Frequencies, percentages, means and standard deviations were used to analyse the socio-demographic characteristics of respondents. Means and standard deviations were used to assess responses to the causes and management of aggression.

## Results

### Socio-demographic characteristics of participants

Approximately 90 clinical staff were invited to take part in the study, and of these, 85 completed the questionnaire, equivalent to 78% of the total number of staff in the three units. Unit 3 had the highest level of participation, followed by units 1 and 2 respectively. Almost two-thirds of respondents were female. The mean age of respondents was 43 years, ranging from 24 to 62 years. About half were born in Australia, just over 40% in Asian countries, and just under 10% in western European countries. Most respondents (88%) were registered and enrolled nurses, while the remainder were medical and allied health staff (Table 1).

**Table 1:** Socio-demographic characteristics of participants (N = 85)

				**n**	**%**
*Gender*	Male			29	34.1
	Female			56	65.9
*Occupation*	Registered nurse			52	61.1
	Enrolled nurse			23	27.1
	Psychiatrist/Allied health			10	11.8
*Unit*	1			26	30.6
	2			22	25.9
	3			37	43.5
*Country/region of birth*					
Australia				40	47.6
Asia				36	42.9
Western Europe				8	9.5
*Age* (years) (n = 81)		M	SD	Min	Max
		43.1	11.3	24	62

### Causes and management of patient aggression

The results are presented in two main sections: (i) causes of aggression, and (ii) management of this behaviour.

### Causes of aggression

#### Internal factors

The overall mean score for internal causes of aggression was 2.6 (SD = 0.4), suggesting respondents had a tendency to disagree that factors directly attributable to the patient contributed to this type of behaviour. Specifically, they perceived aggression was associated with mental illness, and particular types of patients were prone to aggression. They also indicated aggressive behaviour was preventable, but tended to disagree that such behaviour resolved of its own accord if the patient was left alone (Table 2).

**Table 2:** Means and standard deviation (SD) of beliefs about the cause of aggression

**No.**		**Mean**	**SD**
*Internal causative factors*			
5	It is difficult to prevent patients from becoming violent or aggressive.	2.5	0.8
7	Patients are aggressive because they are ill.	2.2	0.7
9	There appear to be types of patients who frequently become aggressive towards staff.	2.2	0.8
12	Patients who are aggressive towards staff should try to control their feelings.	3.1	1.0
17	Aggressive patients will calm down automatically if left alone.	2.6	0.7
*External causative factors*			
1	Patients are aggressive because of the environment they are in.	2.4	0.9
19	Restrictive care environments can contribute towards patient aggression and violence.	1.8	0.5
30	If the physical environment were different, patients would be less aggressive.	1.9	0.9
*Situational/interactional causative factors*			
2	Other people make patients aggressive or violent.	2.2	0.7
3	Patients commonly become aggressive because staff do not listen to them.	2.5	1.0
8	Poor communication between staff and patients leads to patient aggression.	2.0	0.7
23	Improved one to one relationships between staff and patients can reduce the incidence of patient aggression and violence.	1.5	0.6
26	It is largely situations that contribute towards the expression of aggression by patients.	2.3	0.7
*Cultural/gender causative factors*			
4	Gender mix of staff on the wards is important in the management of aggression.	1.9	0.9
6	Patients from particular cultural groups are more prone to aggression.	2.2	0.9
10	Cultural misunderstandings between patients and staff can lead to aggression.	1.9	0.8

Rating scale: 1 = strongly agree, 2 = agree, 3 = disagree, 4 = strongly disagree.

#### External factors

The overall mean score for external causes was 2.0 (SD = 0.4), indicating respondents tended to agree that environmental factors in the units were influential in causing aggression. Restrictive care environments, such as locked wards, were perceived as contributing to aggression. Likewise, respondents were in agreement that if the physical environment was better patients would be less likely to be aggressive (Table 2).

#### Situational/interactional factors

The overall mean score for situational/interactional causes of aggression was 2.1 (SD = 0.5), suggesting respondents were in agreement that factors in the immediate situation, including the way staff communicated with patients, contributed to this type of behaviour. In particular, the respondents were in agreement that patient aggression occurred because of the influence of others. Patients were also more likely to become aggressive because of poor patient-to-staff communication. However, there was agreement-to-disagreement that this form of challenging behaviour was attributable to staff failing to listen to patients (Table 2).

#### Cultural/gender factors

The overall mean score of 2.0 (SD = 0.7) suggested respondents were in general agreement with the statements that cultural and gender influences contributed to the initiation of aggression in units. In particular, there was agreement patients’ cultural background and cultural miscommunications between patients and staff contributed to the onset of aggression. Likewise, there was agreement gender mix of staff was an important consideration in dealing with aggression (Table 2).

### Management of aggression

The overall mean score for the management of this type of behaviour was 2.3 (SD = 0.3), indicating respondents had a tendency to agree with the statements about how to respond to these challenging situations (Table 3). Generally, there was agreement patient aggression could be dealt with more effectively in the units. In particular, there was agreement medication was useful for treating aggression and it should be used more frequently with patients who displayed this behaviour. However, they also responded that in some circumstances medication contributed to instances of aggression.

**Table 3:** Means and standard deviation (SD) of beliefs about the management of aggression

**No.**		**Mean**	**SD**
*Management: General*			
11	Different approaches are used on this ward to manage patient aggression and violence.	1.7	0.8
24	Patient aggression could be handled more effectively on this ward.	2.1	0.8
*Management: Use of medication*			
16	Medication is a valuable approach for treating aggressive and violent behaviour.	2.3	0.9
25	Prescribed medication can in some instances lead to patient aggression and violence.	2.2	0.6
28	Prescribed medication should be used more frequently to help patients who are aggressive and violent.	2.4	0.9
*Management: Use of seclusion*			
13	When a patient is violent, seclusion is one of the most effective approaches to use.	2.7	0.9
15	The practice of secluding violent patients should be discontinued.	3.0	0.8
27	Seclusion is sometimes used more than necessary.	2.5	0.9
*Management: Restraint*			
14	Patients who are violent are often restrained for their own safety.	2.5	0.9
21	Physical restraint is sometimes used more than necessary.	2.4	0.9
*Management: Non-physical methods*			
18	Negotiation could be used more effectively when managing aggression and violence.	1.9	0.7
20	Expressions of aggression do not always require staff intervention.	2.5	0.8
22	Alternatives to the use of containment and sedation to manage patient violence could be used more frequently.	2.1	0.7
29	The use of de-escalation is successful in preventing violence.	1.7	0.6

Rating scale: 1 = strongly agree, 2 = agree, 3 = disagree, 4 = strongly disagree.

The findings indicated agreement-to-disagreement that seclusion was one of the most effective ways for dealing with this form of behaviour, restraint and seclusion were sometimes used more often than necessary, and patients were usually restrained for their own safety. Moreover, there was disagreement with the statement that the practice of seclusion should be discontinued.

There was general agreement person-centred alternatives to containment and sedation, such as negotiation and de-escalation, could be used more effectively to prevent and manage aggression. However, there was agreement-to-disagreement about whether or not staff should always intervene in situations when patients were aggressive.

## Discussion

The findings of our study provide a valuable insight into the attitudes of clinical staff respondents toward the contentious issue of the causes and management of aggression in acute old age psychiatry inpatient settings. In relation to the causes, overall, the respondents tended to disagree that internal or direct patient related factors were contributing influences. However, they perceived aggression was related to mental illness, and some patients were more susceptible to aggression than others. These findings suggest that while the respondents were less likely to attribute blame to patients for aggression, they perceived those with certain conditions were more susceptible to these forms of behaviours. This view is consistent with some literature suggesting patients with persecutory delusions [5,16] and impulsivity [17] are particularly prone to aggression; the risk is greater during the acute phase of psychotic illness; and thought disorder, impairment of neuropsychological functioning, disorganized behaviour and substance misuse, are lesser contributing factors [2,13,43-45].

The respondents in the current study also indicated socio-demographic influences; in particular, cultural consideration had contrasting effects on the onset of aggression. They felt patients from some cultural backgrounds were more susceptible to aggression than others, and cultural misinterpretations between staff and patients contributed to this behaviour. There are contrasting findings in the limited psychiatric literature examining the influence of cultural background on aggression. Depp [46] found black patients are overrepresented in the striking role whereas white patients are more likely to be in the non-striking role. However, James et al. [18] reported there were no statistical differences between the cultural backgrounds of violent and non-violent patients. Therefore, in the present study cultural misinterpretations may be attributed to poor staff-to-patient interactions [9,10], which can lead to frustration and this, in turn, increases the likelihood of aggression [1]. They may also be due to cultural misunderstandings wherein some illness related behaviours, such as aggression and loud speech, are perceived as abnormal by clinicians but may be perfectly acceptable within a particular patient’s culture [47].

The respondents agreed that gender mix of staff on wards was helpful in the prevention of aggression. There are contrasting findings in the literature, however, about the influence of staff gender on patient aggression. Daffern et al. [48], in a 6-month review of episodes of patient aggression in a forensic psychiatric hospital, found no statistically significant relationship between the gender ratio of staff and aggression. However, a review of ecological factors influencing inpatient psychiatric unit violence, by Hamrin et al. [8], presented conflicting findings about staff gender and aggression; with some studies concluding male staff were at greater risk of being recipients of violence, whereas other studies reported females staff were the most common recipients of violence.

The respondents in the present study tended to agree that external and situtational/interactional (or situational and contextual) influences contributed to the onset of aggression, including the use of restrictive environments such as locked wards. They also perceived if the physical environment was improved patients would be less prone to aggression. These findings can be explained by external and situational/interactional influences, such as restrictive environments, which increase the likelihood of frustration and the possibility of aggression [1]. The findings also accord with other studies that report the physical characteristics of the ward environment, such as irritating noise, lack of privacy, restriction in liberty [12,43,44,49], and lack of activities [11], contribute to aggression.

There was agreement in the current study that poor staff-to-patient communication contributed to the onset of aggression. Whittington and Wykes [50], in a United Kingdom study of inpatient aggression, also reported the influence of aversive stimulation by staff, such as physical contact, frustration, demands patients participate in activities, and critical comments. It can also be extrapolated that poor staff-to-patient communication leads to frustration [1,9,10], which, in turn, may culminate in patient aggression.

Regarding the management of patient aggression, the respondents were in agreement this behaviour could be managed more successfully, and a person-centred approach could be used more often as well as judicious use of medication. These findings can be explained, whereby external stimuli, such as person-centred and careful medication-use techniques [10,11], could have a moderating influence in reducing the likelihood of aggression [1].

The findings of the present study also indicated restraint and seclusion were used more than necessary in the management of aggression, and there was disagreement about whether the practice of seclusion should be stopped. The preference by staff to retain the option to use seclusion has also been reported in United Kingdom studies by Duxbury and Whittington [37] and Foster et al. [51]. Possible explanations for the somewhat contradictory findings in the current study are person-centred and pharmacological approaches are perceived as insufficient to deal with all instances of aggression. Another explanation is staff support for these containment practises is affected adversely by workplace situational and contextual and broader cultural influences and is resistant to change. To illustrate, support for such practises may be lower in countries where these approaches are uncommon but higher in countries where they are adopted more often, such as Australia [21,23,52]. Furthermore, the current study’s findings highlight a dichotomy between government [25,26], service user [19,52] and carer groups [19] that advocate the abolition of seclusion and the views of coalface face clinicians confronted with this type of challenging behaviour.

### Limitations and strengths

While a representative sample of 78% of clinical staff was obtained, the data may not accurately reflect the views of all such staff because those working at weekends and at night were not included. This could be addressed in a future study by recruiting respondents throughout the 24-hour, 7-day spectrum. Next, as the sample was derived from three inpatient units within a single mental health service, this limits the ability to infer from this sample to the general population of clinical staff working in other acute old age psychiatry inpatient units. Recruiting respondents from a wider range of mental health services could rectify this in a subsequent study. Finally, the sample only included the views of clinical staff; current and former inpatients were considered for inclusion but were not deemed well enough by clinical staff to provide consent.

## Conclusions

Our survey provides a valuable insight into the contentious issue of attitudes toward the causes and management of aggression, and contributes to the limited body of research about this issue in old age psychiatry inpatient settings. Consideration of attitudes is important because there is a direct relationship between attitudes and the types of measures that are used to manage this challenging behaviour. It can be inferred from the findings of this study that a broad approach to prevention and management of aggression — one that addresses socio-demographic and interpersonal as well as situational and contextual influences — needs to be adopted. They findings also show clinical staff possess contrasting attitudes about the most effective and acceptable ways to prevent and manage this challenging behaviour; in particular, there were mixed views about whether the practice of seclusion should cease.

Therefore, measures taken to address prevention and management should also focus on these contrasting attitudes. Overall, strategies to prevent and manage aggression in acute old age psychiatry inpatient settings have implications for the overall quality of care; in particular, patient charactristics, inpatient environment, the way staff interact with patients, cultural sensitivity and gender mix of staff, and consideration of appropriate ways to manage aggression such as the adoption of person-centred alternatives to restraint and seclusion.

## Competing interests

The authors declare that they have no competing interests.

## Authors’ contributions

TMcC had a major role in the design of the study, undertook some data collection, carried out the data analysis, and had a major role in writing the paper. JB had a major role in the design of the study, and had a key role in writing the paper. EMC had a major role in the design of the study, and had a major role in writing the paper. All authors have approved the final draft.

## Pre-publication history

The pre-publication history for this paper can be accessed here:

http://www.biomedcentral.com/1471-244X/14/80/prepub
